# Evaluation of Emergency Response Capacity of Urban Pluvial Flooding Public Service Based on Scenario Simulation

**DOI:** 10.3390/ijerph192416542

**Published:** 2022-12-09

**Authors:** Yongling Zhang, Miao Zhou, Nana Kong, Xin Li, Xiaobing Zhou

**Affiliations:** School of Emergency Management, Henan Polytechnic University, Jiaozuo 454000, China

**Keywords:** urban pluvial flooding, scenario simulation, emergency response, capacity evaluation, accessibility, GIS

## Abstract

The evaluation of emergency response capability under different pluvial flooding scenarios is an essential approach to improve the emergency response capability of flood disasters. A new evaluation method of emergency response capacity of urban public services is proposed based on urban pluvial flooding scenario simulation. Firstly, inundation area and depth under different pluvial flooding scenarios are simulated based on the SCS-CN model. Following that, space densities of all indicators include inundation area and depth, road network and the emergency public service institutions. Then, the indicator weight is determined by the combined weighting method of entropy weight and coefficient of variation. Finally, the emergency response capacity index (of each pixel) is calculated based on the graph stacking method. Taking Erqi District, Zhengzhou City as an example, the emergency response capacity of public service under different urban flooding scenarios is evaluated. The results show that the spatial distribution difference of public service emergency response capacity in Erqi District, Zhengzhou City is obvious, and with the increase of the precipitation return period, the high value area of public service emergency response capability decreases gradually and the low value area increases gradually. This method takes into account the specific urban flooding scenario and the layout of public service institutions and road networks that have strong practicability. the results of the evaluation can provide a reference for the construction of urban flood emergency response capacity and provide support for emergency decision-making.

## 1. Introduction

Affected by global climate change and the process of urbanization, urban flood disasters caused by extreme precipitation events seriously threaten urban public safety and the sustainable development of the economy and society [1]. Assessment reports of the Intergovernmental Panel on Climate Change (IPCC) point out that climate change will bring more extreme weather events, and the intensity, frequency and duration of precipitation will continue to increase. Pluvial flooding has become the focus of public and academic concern [2]. East China is a typical East Asian monsoon region, where the precipitation is concentrated in summer and extreme precipitation often occurs, which easily causes floods. On the other hand, with the deepening of China’s reform and opening up and the development of social economy and the acceleration of the urbanization, big cities and megacities are emerging. With the expansion of urban ground hardening areas, the infiltration capacity decreases, and the surface net discharge and runoff intensity increase, which is more likely to cause flood disasters [3]. In recent years, more than 62% of large and medium-sized cities in China have experienced severe urban pluvial flooding, which has had a serious impact on the economy and society. For example, on 18 July 2007, the main urban area of Jinan suffered severe pluvial flooding disasters, resulting in most sections of traffic being paralyzed, causing more than 30 people to die, about 330,000 people affected and direct economic losses of about CNY 1.32 billion. On 21 July 2012, Beijing was severely affected by the “7201321” heavy rain, which caused severe urban waterlogging and disrupted some road traffic, resulting in 79 deaths, 1.602 million people affected and direct economic losses of CNY 11.64 billion. In July 2016, the urban flood disaster caused by a rainstorm in Wuhan resulted in 14 deaths, with 757,000 people affected, some roads paralyzed and direct economic losses of CNY 2.265 billion. From 17 to 23 July 2021, Zhengzhou suffered a rare rainstorm in history, with the cumulative area precipitation of 534 mm, which was the most extensive and strongest rainstorm process since the inception of meteorological observation records in Zhengzhou. The heavy rainstorm caused serious floods, with city traffic disruption, resulting in 380 deaths or missing and direct economic loss of CNY 40.9 billion. According to the ‘Henan Zhengzhou ‘7·20’ heavy rainstorm disaster investigation report’ submitted by the State Council’s Disaster Investigation Team, the lack of emergency response capacity has become one of the important factors causing casualties missed by the rainstorm disaster in Zhengzhou. Therefore, strengthening the city, especially megacities’ pluvial flood disaster management and emergency capacity building, has not only become a major practical need, but also has received a lot of attention from the academic community. For example, Zhang et al. [4], Zhong et al. [5] and Chen et al. [6] conducted theoretical discussions on the demand, reserve and distribution of emergency supplies. Slobodan et al. [7] and Liu et al. [8] conducted necessary research on public flood risk perception and emergency evacuation.

Urban pluvial flooding disaster management requires not only flood mitigation and prevention, but also rapid response when flood disasters occur, and in particular, rapid and efficient emergency response is the most direct and effective measure to reduce urban flood disaster losses [9,10]. The accessibility of roads is affected by the traffic disruption caused by urban flood disasters, which reduces the emergency response capacity of public service facilities such as medical, firefighting and public security [11]. Researchers have begun to study the emergency response capacity of public service facilities under flood conditions by combining flood numerical simulation with GIS analysis technology. Shi et al. [12] and Coles et al. [13] pointed out that under the flood scenario, some road networks become paralyzed, traffic is interrupted and emergency vehicles cannot pass, resulting in the scope of emergency medical service space being significantly less than usual. Green et al. [14] showed that the impact of pluvial flooding on urban emergency response accessibility was more severe than that of river floods on the same scale. The function of emergency response is mainly affected by the location of emergency facilities and the function of traffic network; moreover, the accessibility of public service emergency response to urban floods decreases with the increase of precipitation return periods [15]. In several other evaluations of emergency response capability studies, such as the evaluation of comprehensive emergency capacity in urban flood disasters in Zhengzhou City by Li et al. [16] and evaluation of emergency response capability in China by Wang et al. [17], emergency response capability indices were developed based on social, economic, environmental and environment indicators, which come from social-economic demographic statistics data. None of above-mentioned studies have incorporated specific simulations of flooding in the assessment, which has limited the use to develop emergency planning and emergency capacity building. The advantages of incorporating flood simulation into emergency response capacity assessments are that it can clarify the emergency response capacity of specific flood scenarios and enhance the pertinence and practicability of emergency response capacity assessments.

In view of this, this paper proposed a new evaluation method for emergency response capacities of urban public services based on urban pluvial flooding scenario simulation. Taking the Erqi District of Zhengzhou City, a megacity in central China as an example, the emergency response capacity of public services (medical, firefighting and public security) is assessed in different urban pluvial flooding scenarios. Firstly, Chicago design storms and the Zhengzhou rainstorm formula were used to calculate of 50-year, 100-year and 500-year precipitation scenarios. Secondly, the SCS-CN model was used to simulate the inundation area and inundation depth under 50-year, 100-year and 500-year precipitation scenarios. Then, the emergency response capacity of public services (medical, public security and firefighting) was again assessed by GIS spatial analysis techniques. Finally, the results of emergency capability evaluation are analyzed, and the concise countermeasures are put forward. This study enriches and develops the theory and methodology of emergency response capacity assessments. the results could provide decision support for disaster prevention and mitigation, and the emergency response and optimization layout of public service facilities.

## 2. Study Area and Data Sources

### 2.1. Study Area

Zhengzhou is one of the megacities in central China with a permanent population of more than 10 million, and is the political, economic and cultural center of Henan Province. The Erqi District is one of the central urban areas of Zhengzhou City, located between 113°30′ E~113°41′, 34°36′ N~34°46′. The terrain is high in the southwest and low in the northeast. The average altitude of the whole area is 177.9 m.

**Figure 1:**
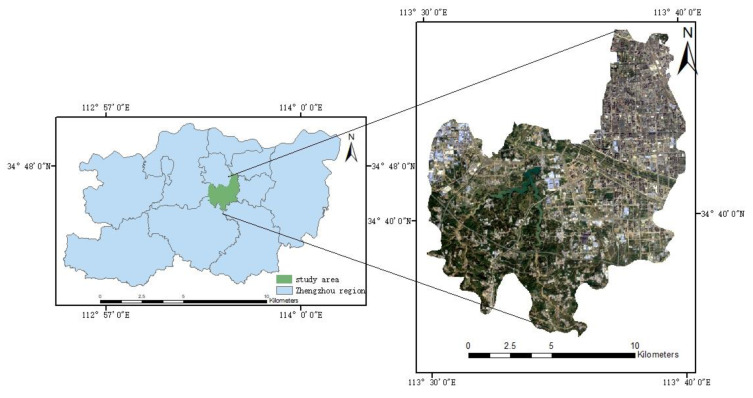
Location of the study area.

Erqi District has a warm temperate continental monsoon climate, precipitation is mainly concentrated from June to August and the average annual precipitation is 632 mm. The total area of district is 156.2 square kilometers. The permanent population is 1.0632 million, and has an urbanization rate is 91.05%. The location of the study area is shown in Figure 1.

### 2.2. Data Sources

**Figure 2:**
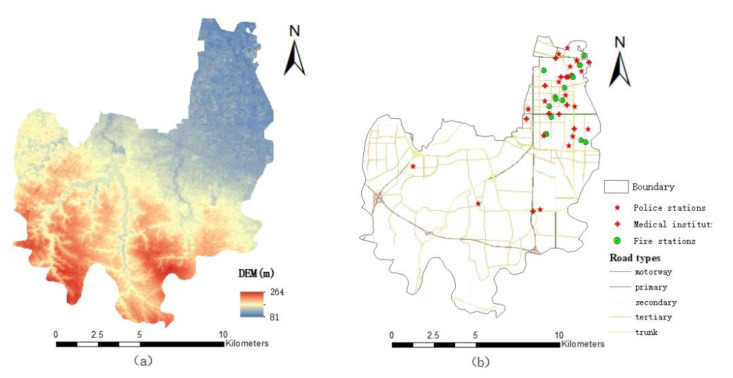
Schematic of study area: (**a**) Digital elevation model (DEM); (**b**) the location of Public Service Organization and Road types.

The research data mainly include terrain elevation data, precipitation data, road network data, remote sensing data and emergency public service department data. Precipitation data of different precipitation return periods in the Erqi District of Zhengzhou City is based on Chicago design storms, remote sensing data (Landast-8 OLI_TIRS 30M) and digital elevation model (GDEMV3 30M) data from the Geospatial Data Cloud (www.gscloud.cn, (accessed on 10 August 2022)). Regarding road network vector data from the OpenStreetMap website (https://www.openstreetmap.org, (accessed on 10 August 2022)), with reference to Zhengzhou urban planning and management technical regulations, the urban road is divided into five levels. In this paper, medical institutions (120 first-aid), public security (110 police) and firefighting institutions (119) are selected as public service institutions for urban flood emergency response. There are 12 public medical institutions at grade II and above, 18 public security organs at or above the level of the police station and 17 fire stations in the study area, excluding community police offices, community health stations and other basic units without an emergency response capacity. The location information of main urban public service departments is extracted from Zhengzhou Municipal People’s Government and Zhengzhou Municipal Health Commission. Figure 2 shows the elevation of the study area (a) and the location of public service institutions (b).

## 3. Method

Based on the rainfall data obtained from the rainstorm intensity formula of Zhengzhou City, the pluvial flooding scenarios with return periods of 50 years, 100 years and 500 years were constructed to determine the cascading impact of flooding on the emergency response capability of public service institutions. 

The Chicago design storms and Zhengzhou rainstorm formula are used to simulate the precipitation in different precipitation return periods, combined with the SCS-CN model and land-use type to calculate the actual precipitation of different precipitation return periods. Based on the idea of dichotomy, the equal volume method of GIS is used to simulate the depth and area of flood inundation [18]. Then, using the GIS raster space analysis and mapping function, the corresponding raster data layer is generated. In order to avoid the defect of the single weight calculation method, the indicator weight is determined by the combination weighting method of entropy weight and coefficient of variation, and the layer is superimposed to evaluate the emergency response ability under different pluvial flooding scenarios in the Erqi District.

### 3.1. Precipitation Data

Combined with the maximization characteristics of urban waterlogging hazards, short duration precipitations with return periods of 50 years, 100 years and 500 years are simulated by the equation of the Zhengzhou rainstorm formula, which was developed by the Central South China Municipal Engineering Design Institute and the Chicago design storms’ precipitation peak coefficient of 0.4, time interval of 1 min and total precipitation duration of one hour [19,20].

(1) $$q=\frac{3037\;\left(1+0.892\mathrm{lgp}\right)}{{\left(t+15.1\right)}^{0.824}}$$

where q is precipitation intensity, mm/min; t is the duration of precipitation, min; p is the return period, year.

### 3.2. SCS-CN Model

The SCS-CN model is a precipitation runoff model developed by the water and Soil Conservation Service of the United States Department of Agriculture, which considers regional underlying surface factors and meteorological factors, and reflects the influence of different soil types and land use types on runoff [21,22,23]. The SCS-CN model is used to calculate runoff, and the final calculation equation is as follows:

(2) $$Q=\left\{\begin{array}{l} \frac{(P\;-\;I_{a}{)}^{2}}{P\;-\;I_{a}\;+\;S}\;\;\;\;P>I_{a}\\ 0\;\;\;\;\;\;\;\;\;\;\;\;\;\;\;\;P\leq I_{a} \end{array}\right.$$

where Q is the direct runoff (mm); P is the total precipitation (mm); S is the potential maximum retention (mm); $I_{a}$ = λS, λ is the initial loss rate (0.1 ≤ λ ≤ 0.3), which is mainly affected by geological and meteorological factors. Based on the existing studies, the λ value is 0.2 [24,25]. The calculation formula of S value is:

(3) $$S=\frac{25400}{\mathrm{CN}}-245$$

**Figure 3:**
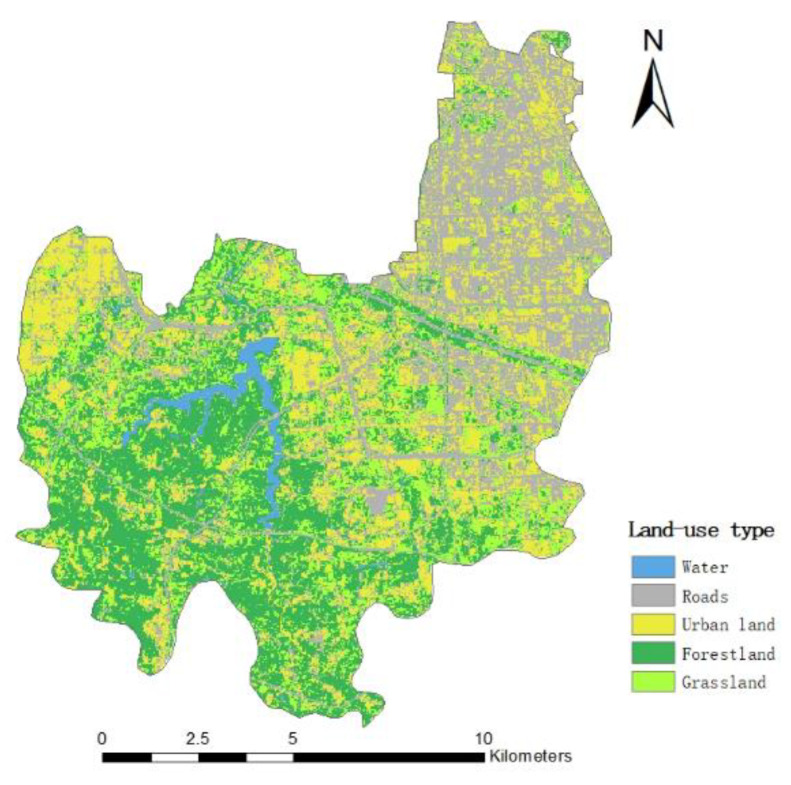
Land Use Types in Erqi District of Zhengzhou City.

CN is curve number, which is related to the underlying surface, land use, hydrological conditions and soil conditions [26]. The higher CN value, the higher the runoff capacity. Based on the maximum likelihood method, ENVI software is used to supervise and classify remote sensing images into five categories: forestland, grassland, water, urban land and roads; the overall classification accuracy was 84.9% and the specific distribution is shown in Figure 3. According to the table of CN values in Chapter 9 of the National Engineering Manual, the CN value of the Erqi District of Zhengzhou City under normal conditions (AMCII type) is determined. According to the literature, the soil type in this area is mainly sandy clay loam [27,28,29]. The corresponding CN value of Group C and the calculated data is shown in Table 1.

The region was extracted by the watershed function of the hydrologic analysis module. Since the land-use types in each region are different, the area weighted average method is used to calculate the CN value; namely, the proportion of land-use type in cities and their respective CN values are weighted [30]. The calculation formula is as follows:

(4) $$\mathrm{CN}={\;f}_{1}\times {\mathrm{CN}}_{1}+f_{2}\times {\mathrm{CN}}_{2}+f_{3}\times {\mathrm{CN}}_{3}+f_{4}\times {\mathrm{CN}}_{4}+f_{5}\times {\mathrm{CN}}_{5}$$

where $f_{1}\sim f_{5}$ represent the area proportion of different land-use types, and CN, ${\mathrm{CN}}_{1}\sim {\mathrm{CN}}_{5}$ represent the composite CN value and the CN value of different land-use types, respectively.

In a runoff analysis, the drainage capacity of the city should be considered. According to the technical regulation and actual situation of urban planning and management in Zhengzhou City, the actual drainage capacity of the Erqi District drainage network is set as once a year (36 mm/h), and three scenarios with precipitation return periods of 50 years, 100 years and 500 years are selected. The boundaries of these catchments divide the study area according to flow direction, flow accumulation (flow accumulation is calculated from flow direction data), flow chain including flow sequence, etc. Based on SCS-CN model, the runoff of each catchment area under the three scenarios is obtained. The product of the difference between runoff and water discharge and the area of the study area is the theoretical value of the total amount of waterlogging in the area [31].

(5) $$W\;=\left(Q\;-\;V\right)\times \;S$$

where W is waterlogging volume; Q is the runoff volume; V is water discharge (36 mm/h); S is the catchment area. ENVI software is used to preprocess remote sensing images (including radiometric calibration, atmospheric correction and geometric correction, etc.), and the maximum likelihood method is used for supervised classification. The accuracy of land classification is calculated by constructing a confusion matrix. The original DEM data is filled with depressions to eliminate abnormal elevation values, and the D8 algorithm of the GIS hydrological analysis module is used to identify the watershed, confluence river network, waterlogging point, divided catchment area and calculated waterlogging volume of each sub-catchment area. Based on the idea of dichotomy, the local equal volume method of GIS is used to simulate the inundation area and depth, and the catchment area of the whole region is regarded as a non-source flood. The points with elevations lower than the water level are all submerged areas, and the precipitation, runoff and waterlogging amount with three precipitation return periods of 50, 100 and 500 years are obtained.

### 3.3. Evaluation of Flood Emergency Response Capacity

#### 3.3.1. Selection and Processing of Indicator Data

Taking the Erqi District of Zhengzhou City as the research area, based on the existing rescue forces, the emergency response capacity of flood disasters in the Erqi District is evaluated. Following the selection principle of indicators, four influencing factors including flood disaster inundation depth, inundation area, road density and density of emergency public service institutions are selected. GIS spatial analysis is a spatial data analysis technology based on the location and morphological characteristics of geographic objects, including overlay analysis, buffer analysis, terrain analysis, density analysis, raster calculation and other modules. The Raster Calculator in GIS spatial analysis technology to obtain the indicator value of each grid and generate grid data layer was used. The extremum method is used to make dimensionless treatments for each indicator, which is divided into positive indicators and negative indicators, different types of indicators are treated according to different formulas and the calculation formula is as follows:

(6) $$\mathrm{positive}\;\mathrm{indicators}:\;x_{ij}=\frac{x_{j}-x_{max}}{x_{max}-x_{min}}$$

(7) $$\mathrm{negative}\;\mathrm{indicators}:\;x_{ij}=\frac{x_{max}-x_{j}}{x_{max}-x_{min}}$$

where $x_{ij}$ is the normalized variable value; $x_{j}$ is the original data; $x_{max}$ is the maximum value; $x_{min}$ is the minimum.

#### 3.3.2. Combination Weighting Method

(1) Entropy Weight Method. The entropy weight method is an objective weight assignment method, which mainly determines the weight of the indicator according to the dispersion degree of the evaluation indicator [32], and the calculation formula is as follows:

(8) $$w_{i}=\frac{1-H_{i}}{m-\sum_{i=n}^{m}H_{i}}$$

where $H_{i}=-\frac{1}{lnn}\sum_{j=1}^{n}f_{ij}lnf_{ij}$ is called information entropy, and $f_{ij}=\frac{b_{ij}}{\sum_{j=1}^{n}b_{ij}}$.

(2) variation coefficient method. The coefficient of variation method calculates weights from the information contained in the indicators, is an objective weighting method that effectively reflects the relative importance of the evaluation indicators and the calculation formula is as follows:

(9) $$C_{i}=\frac{\sigma_{i}}{x_{i}}$$

(10) $$V_{i}=\frac{C_{i}}{\sum_{i=1}^{n}C_{i}}$$

where $C_{i},\sigma_{i},x_{i}$ and $V_{i}$ are the coefficient of variation, mean squared error and mean and weight values of the indicator, respectively.

(3) Combination weight. Assuming that the importance of the two weighting methods is the same, the combined weighting method based on entropy weight and the coefficient of variation method is constructed to realize the complementary advantages of objective weighting methods, and the formula can be expressed as follows:

(11) $$w=w_{i}\times 0.5+V_{i}\times 0.5$$

## 4. Results and Discussion

### 4.1. Flood Inundation Analysis

**Figure 4:**
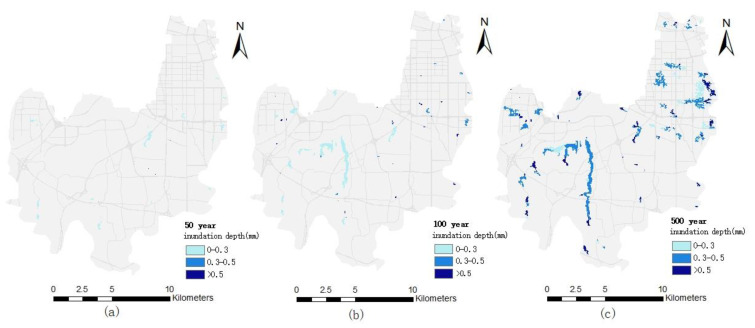
Simulation map of inundation area and depth under different pluvial flooding scenarios in the Erqi District: (**a**) the 50-year pluvial flooding scenario; (**b**) the 100-year pluvial flooding scenario; (**c**) the 500-year pluvial flooding scenario.

The simulation results of the inundation area and inundation depth under different pluvial flooding scenarios in the Erqi District of Zhengzhou City are shown in Figure 4. With the increase of the precipitation return period, rainfall and runoff increase gradually and the flood accumulates along the river course and flows to the middle and lower reaches, where the terrain is lower. The depth and area of inundation will increase as water levels increase in tributaries and low-lying areas within the region.

Under the 50-year pluvial flooding scenario, the inundation area in the study area is 0.98 km^2^, and the depth of inundation is almost always below 30 cm. The roads are accessible and public service rescue vehicles can pass through.

Under the 100-year pluvial flooding scenario, the inundated area increases significantly to 3.37 km^2^, but the area of inundation depth over 30 cm is small, only 0.43 km^2^, which is mainly distributed around Jiangang Reservoir and part of Lianyun Road. According to the urban water closure standards (when the road reaches 25–35 cm), the passage of rescue vehicles will be affected in some road sections, which will delay emergency rescue to a certain extent.

Under the 500-year pluvial flooding scenario, the inundated area is further increased to 11.84 km^2^, of which the inundated area with a depth of 30–50 cm is 3.97 km^2^ and the inundated area with a depth of more than 50 cm is 5.89 km^2^. The inundated area is relatively scattered, which shows a zonal distribution around Jiangang Reservoir and on both sides of the Jinshui River and irregular lumps or spots in other areas. Some sections of the low-lying Lianyun Road and Changjiang Road have accumulated water of more than 50 cm and even more than 1 m, which can seriously affect the passage of emergency rescue vehicles.

### 4.2. Evaluation of Emergency Response Capability

**Figure 5:**
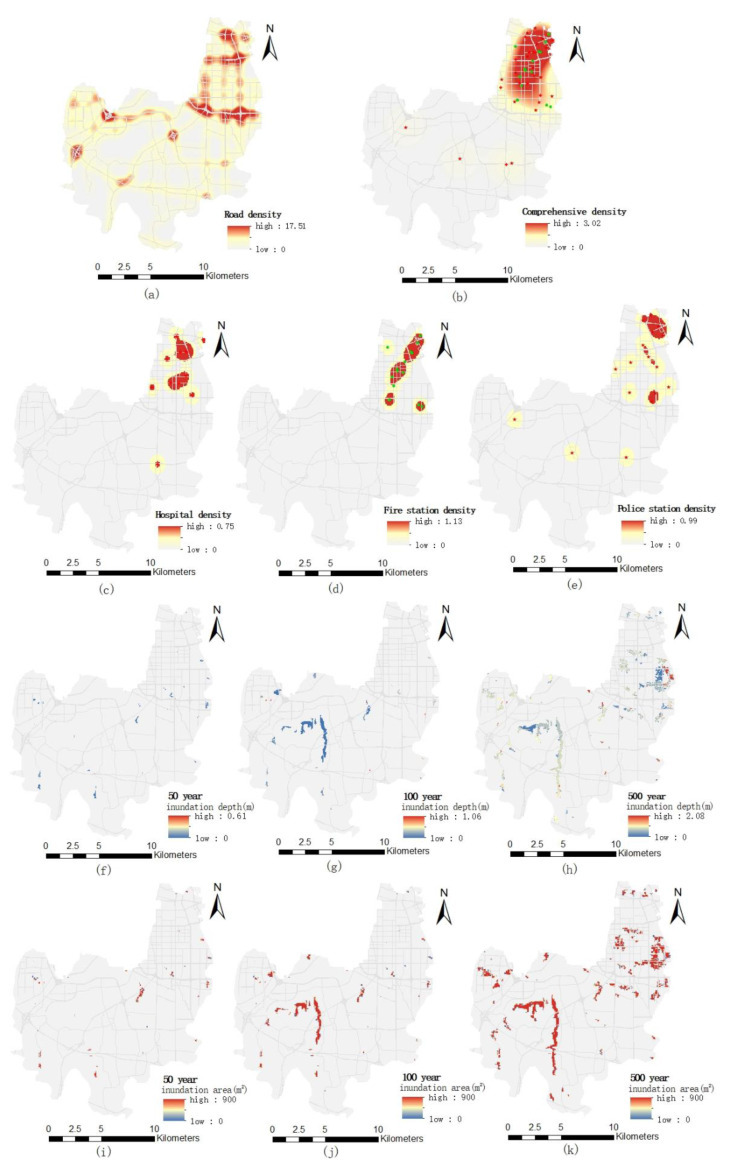
Spatial distribution of emergency response capability evaluation indicators of public service institutions: (**a**) road density; (**b**) comprehensive density of public service institutions; (**c**) hospital density; (**d**) fire station density; (**e**) police station density; (**f**) inundation depth under 50-year pluvial flooding scenario; (**g**) inundation depth under 100-year pluvial flooding scenario; (**h**) inundation depth under 500-year pluvial flooding scenario; (**i**) inundation area under the 50-year pluvial flooding scenario; (**j**) inundation area under the 100-year pluvial flooding scenario; (**k**) inundation area under the 500-year pluvial flooding scenario.

**Figure 6:**
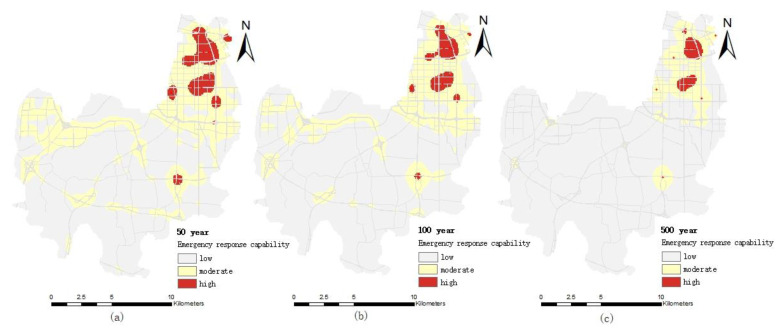
Distribution map of emergency response capacity of medical institutions with different r pluvial flooding scenarios: (**a**) the 50-year pluvial flooding scenario; (**b**) the 100-year pluvial flooding scenario; (**c**) the 500-year pluvial flooding scenario.

**Figure 7:**
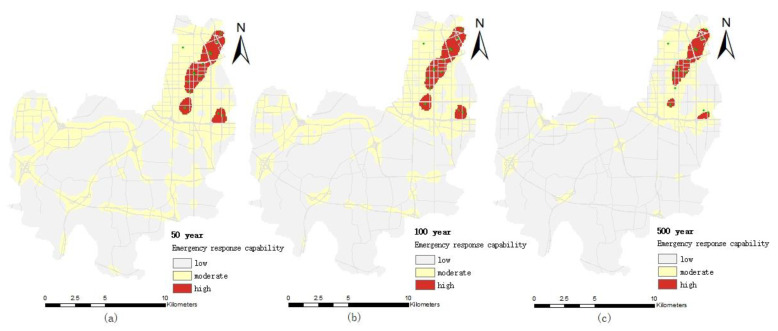
Distribution of emergency response capability of firefighting institutions under different pluvial flooding scenarios: (**a**) the 50-year pluvial flooding scenario; (**b**) the 100-year pluvial flooding scenario; (**c**) the 500-year pluvial flooding scenario.

**Figure 8:**
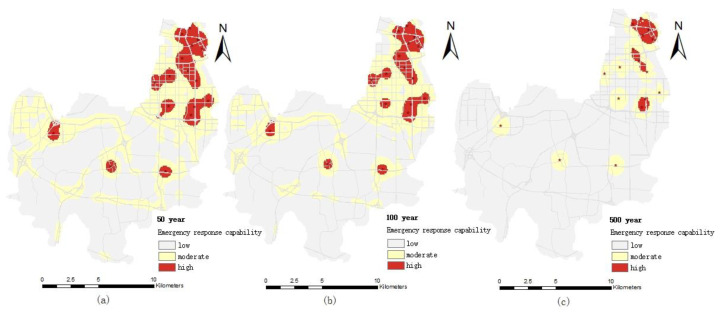
Distribution of emergency response capacity of public security organs under different pluvial flooding scenarios: (**a**) the 50-year pluvial flooding scenario; (**b**) the 100-year pluvial flooding scenario; (**c**) the 500-year pluvial flooding scenario.

**Figure 9:**
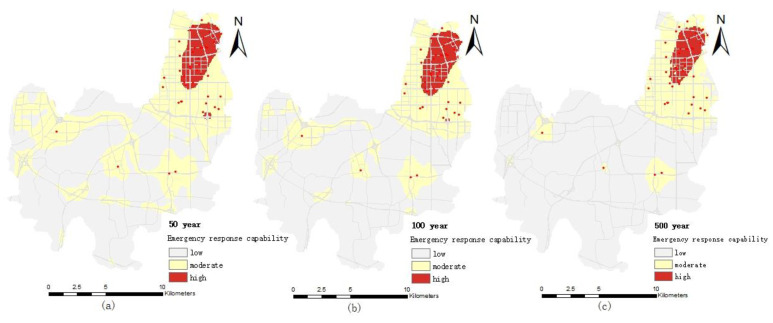
Distribution map of comprehensive emergency response capacity of public service under different pluvial flooding scenarios: (**a**) 50-year pluvial flooding scenario; (**b**) 100-year pluvial flooding scenario; (**c**) 500-year pluvial flooding scenario.

The kernel density analysis tool in the GIS-based spatial analysis obtains the spatial distribution of road network density, medical, public security and firefighting density and their comprehensive density [33] (Figure 5). Then, the emergency response capability and comprehensive emergency response capability of medical, public security and firefighting respectively are evaluated by the overlapping method (Figure 6, Figure 7, Figure 8 and Figure 9). Thereinto, emergency response capacity refers to the emergency response capacity of various medical, public security and fire public service institutions. Comprehensive emergency response capacity is the sum of emergency response capacity of three public service institutions. It is known that the inundation area and inundation depth are negatively correlated with the emergency response ability, whereas the density of public service institutions and roads is positively correlated with the emergency response ability. The longer the precipitation return period, the larger the inundation area, and the deeper the water elevation, the smaller the emergency response ability and the greater the impact. The smaller the road density is, the more traffic is inconvenient, which is not conducive to rescue work and the smaller emergency response capacity.

#### 4.2.1. Emergency Response Capacity of Medical Institutions 

The distribution of the emergency response capacity of medical institutions under different pluvial flooding scenarios is shown in Figure 6. It can be seen from the figure that (1) the emergency response capacity of medical institutions has obvious space differences. The northeast of the Erqi District of Zhengzhou City is obviously higher than other districts in all pluvial flooding scenarios. Because medical facilities are predominantly located in the region, the region’s emergency capacity is higher than other regions. (2) The emergency response capacity of medical institutions is distributed in a patchy multi-center distribution, and shows a decreasing trend from the center to the periphery. The location of medical institutions with medical emergency response ability forms a center with relatively high emergency response ability. The medical emergency response capability gradually declines from the location (center) of medical institutions, and the speed of attenuation depends on the convenience of road traffic and the distance. (3) With the increase of the precipitation return period, the high value area of medical emergency response capability gradually decreases, and the low value area of emergency response capability is gradually expanded. The main reason is that with the increase of the precipitation return period, the inundation area and depth are increased, which affects the accessibility of roads and makes emergency rescue vehicles delayed or inaccessible.

Under the 50-year pluvial flooding scenario, the inundated area and depth of the Erqi District are small, and 97% of the area has no water accumulation. Although some road sections are inundated, but water depth does not exceed 30 cm, it has little impact on medical rescue and basically reflects the distribution of medical emergency response capabilities under normal conditions.

Under the 100-year pluvial flooding scenario, the inundation area and depth increased, as did road length and depth; for example, the length of the road with an inundation depth of 30–50 cm and above 50 cm is 0.9 km and 0.59 km, respectively, which makes it difficult for medical rescue vehicles to pass safely (when the road water reaches 25–35 cm, it will reach the height of the exhaust port, affecting the safe passage of vehicles), resulting in the decrease of the high value area and the median value area of the emergency response capacity and the increase of the low value area.

Under the 500-year pluvial flooding scenario, the inundation area and depth are further expanded, and the length of the inundation road also increases. The length of the inundated road with a water depth of 30–50 cm increases to 15.79 km and the length of roads with a water depth of over 50 cm is 8.09 km. Therefore, the emergency response capacity of medical institutions is significantly lower than that of the 50 years and 100 years pluvial flooding scenarios.

#### 4.2.2. Emergency Response Capability of Firefighting Institutions 

The distribution of the emergency response capacity of firefighting institutions in different pluvial flooding scenarios is shown in Figure 7. It can be seen from the figure that the high value area and most of the median area of firefighting emergency response capacity are all located in the northeast of the Erqi District, because the firefighting institutions in the Erqi District of Zhengzhou City are located in the northeast. Outside the northeast of the Erqi District, except for the median emergency capacity area near major traffic arteries, all the other areas are low value areas. With the increase of the precipitation return period, the changes to the emergency response capacity of firefighting agencies decreases gradually, which is similar to the tendency of the medical emergency response capacity.

Under the 50-year pluvial flooding scenario, the flood inundation depth is shallow and the affected road sections are less, and the layout and distance of firefighting institutions become the main factors affecting the firefighting emergency response capability.

Under the 100-year pluvial flooding scenario, the inundation area and inundation road are obviously increased, and on some parts of roads with water depths of 30–50 cm, only large and medium-sized firefighting vehicles with good wading performance can pass. The distance from firefighting institutions and driving speed of firefighting vehicles become the main factors affecting the firefighting emergency response capability.

Under the 500-year pluvial flooding scenario, the inundation area and inundation depth continue to increase. Inundation area and road length above 50 cm are 5.89 km^2^ and 8.09 km, respectively, and only a small number of large firefighting vehicles can slowly pass (the exhaust pipe is on the top of the cab). The water depth of most sections near the Daxue Middle Road, the intersection of Ganjiang Road and Xiangyun Road in the Erqi District, is more than 80 cm, making it difficult for firefighting emergency services to get to the area in time.

#### 4.2.3. Emergency Response Capability of Public Security Organs

Compared with the spatial distribution of medical and firefighting emergency response capabilities, the emergency response capabilities of public security agencies are higher in the northeast and there are also three high value areas in the central region (Figure 8), mainly because the middle of the Erqi District also has the layout of public security organs. With the increase in the precipitation return period, the emergency response capability of public security organs gradually decreased, showing that the high value area gradually decreased and the low value area gradually increased, which is similar to the medical and firefighting emergency response capabilities. 

The 50-year pluvial flooding did not cause road disruption, although some sections were flooded, but emergency vehicles, especially large vehicles of the public security organ, could still reach all parts of the region. 

Under the 100-year pluvial flooding scenario, although some roads were flooded with deep water (the length of inundation roads with a depth of 30–50 cm and above 50 cm is 0.9 km and 0.59 km, respectively), large vehicles and special vehicles of the public security organ could pass through. However, water depth affects the speed of emergency vehicles, which causes the delay of emergency response time and reduces the emergency response capability. 

Under the 500-year pluvial flooding scenario, the water depth at the Jingguang police station at the intersection of Changjiang Road and Minggong Road police station at Yanhe Road were over 50 cm. Since police stations generally have no large special service vehicles to provide emergency response services in time, the emergency response capability of public security institutions is seriously reduced. Moreover, the inundation length and depth of roads is also a major reason to reduce the emergency response capacity of public security organs.

#### 4.2.4. Comprehensive Emergency Response Capability 

The normalized inundation depth, inundation area, road density and emergency public service organization density layers are overlaid by spatial analysis technology based on GIS. The emergency response capacity of the Erqi District is divided into three levels (high, medium and low) using the natural discontinuity method. The distribution map of the comprehensive emergency response capacity of public services in the Erqi District is obtained (Figure 9).

The regional differences of comprehensive the emergency response capacity under different pluvial flooding scenarios are obvious. The high value area of the comprehensive emergency response capacity is mainly distributed in the northeast of the Erqi District and generally shows a decreasing trend from northeast to southwest. The main reason is that the public service institutions (medical, firefighting and public security) are concentrated in the northeast of the Erqi District, and the density of the road network is higher in this area. Moreover, due to the relatively high terrain in this region, the inundated area and the inundated depth scenario are smaller and lower under the same flood scenario. Similar to the spatial pattern of emergency response capacity of medical, public security and firefighting institutions, with the increase of the precipitation return period, the high value area of the comprehensive emergency response capacity of public services is gradually decreased and the low value area is gradually increased. 

The proportion of high and median emergency response capability in the study area is 6.53% and 32% in the 50-year pluvial flooding scenario flood scenario, respectively. The high and median areas of emergency response capacity in the 100-year rainstorm scenario accounted for 5.29 % and 28%, respectively. The proportion of high and medium value areas of emergency response capacity in the 500-year pluvial flooding scenario is 3.66% and 17%, respectively. With the increase of the precipitation return period, the low value area of emergency response capacity increased significantly, which increased by 4% and 17% in 100-year and 500-year flood scenario, respectively, compared with the 50-year pluvial flooding scenario.

#### 4.2.5. Robustness Test

In order to enhance the reliability of the empirical study, the grey correlation analysis method [34,35] was used to carry out the robustness test by eliminating some indicators.

Firstly, the grey correlation method is used to calculate the correlation degree of the evaluation index. Then, according to the results of correlation degree, some indicators are eliminated, and the grey correlation method is used to calculate again, and the new evaluation results are obtained. Finally, the calculation results of the two grey correlation methods are compared. If the fluctuation of correlation degree is small, the result is robust; otherwise, it is not robust. The results of the two grey correlation analyses are shown in Table 2 and Table 3.

From the above analysis results, it can be seen that the slight fluctuation of some indicators does not lead to the change of the ranking, which passes the robustness test.

## 5. Conclusions and Prospects

### 5.1. Conclusions

Pluvial flooding is one of the major natural disasters in Zhengzhou, and frequent urban pluvial flood events have seriously affected the sustainable development of society in recent years. For example, the Zhengzhou ‘7·20’ heavy rainstorm disaster caused serious losses. Hence, it is urgent to comprehensively strengthen emergency response capacity building. Furthermore, the evaluation of emergency response capacities under different flood scenarios is an important means to comprehensively strengthen emergency response capacities. This paper proposed a new evaluation method for the emergency response capacity of urban public services based on urban pluvial flooding scenario simulation, in which emergency response capabilities of public services were assessed under the different pluvial flooding scenarios in the Erqi District of Zhengzhou city. The main conclusions are as follows: (1) The inundation area and depth are increased with the increase of the precipitation return period, whereas the emergency response ability of public services is decreased with the increase in the precipitation return period. (2) The emergency response capacity largely depends on the location of public services, the operation of public service institutions and road accessibility. The emergency response capacity of public services shows a trend of decreasing outwards from the centre of the public service. (3) The spatial difference of the emergency response capacity in the Erqi District of Zhengzhou City is significant. The high value areas of emergency response capacity are mainly distributed in the northeast, and emergency response capacity shows a decreasing trend from northeast to southwest, generally. The result of research shows that the evaluation method of emergency response capacity based on urban pluvial flooding scenario simulation can objectively evaluate the emergency response capacity under different pluvial flooding scenarios, which provides a new method for emergency response capacity assessment. 

### 5.2. Prospects

Since the number and location of emergency service agencies have a significant impact on the overall emergency response capacity of the city, it is necessary to strengthen the optimal layout of urban emergency public service facilities. Public service departments should be reasonably distributed in the central and southwestern parts of the Erqi District and some public service agencies located in severely inundated areas should be relocated to places with less risk of flood inundation. At the same time, for low-lying areas prone to water accumulation, the construction of municipal pipe network to improve flood discharge capacity should be strengthened.

There are still some shortcomings in this study. Due to the limitation of data acquisition and accuracy, the size of the raster layer is set as 30 m × 30 m when constructing the raster layer. In further study, the accuracy of the research data should be enhanced to improve the accuracy of flood inundation area identification. Flood disasters have a nonlinear impact on the function (connectivity and accessibility) of urban road systems, traffic congestion and rainstorm flood duration, which also affects the emergency response time of public service agencies. The next step will continue to study the accessibility, service scope and optimal path of public service institutions in cyberspace under different pluvial flooding scenarios (distance or time from public service measured along accessible (non-flooded) roads) to strengthen the research on the impact of urban traffic congestion and human travel behavior on the emergency response capacity of public service institutions. In addition, the inundation depth and area of different pluvial flooding scenarios are simulated based on the passive submerged method, without considering the problem of water flow connectivity. In the future, the flood inundation scenario combining non-source floods and source floods and the emergency response capability of public services will be studied.

## Figures and Tables

**Table 1:** Supervision Classification Calculation Results.

Land-Use Type	CN Value	Area/km^2^	Area Ratio
Forestland	70	52.17	0.334
Grassland	79	29.11	0.186
Water	98	2.952	0.019
Urban land	90	55.25	0.353
Roads	94	16.89	0.108

**Table 2:** Correlation result.

Evaluation Item	Correlation Degree	Rank
Road density	0.949	1
Comprehensive density	0.858	4
Inundation area	0.859	3
Inundation depth	0.947	2

**Table 3:** Correlation result after eliminating some indicators.

Evaluation Item	Correlation Degree	Rank
Road density	0.967	1
Comprehensive density	0.884	4
Inundation area	0.885	3
Inundation depth	0.944	2

## Data Availability

Remote sensing data (Landast-8 OLI_TIRS) and digital elevation model data (GDEMV3 30M) can be downloaded for free in the Geospatial Data Cloud (www.gscloud.cn, (accessed on 10 August 2022)). Road network vector data can be downloaded for free in the OpenStreetMap website (https://www.openstreetmap.org, (accessed on 10 August 2022)).
